# Impact of Ergothioneine, Hercynine, and Histidine on Oxidative Degradation of Hyaluronan and Wound Healing

**DOI:** 10.3390/polym13010095

**Published:** 2020-12-29

**Authors:** Katarina Valachova, Karol Svik, Csaba Biro, Maurice N. Collins, Rastislav Jurcik, Lubomir Ondruska, Ladislav Soltes

**Affiliations:** 1 Centre of Experimental Medicine, Institute of Experimental Pharmacology and Toxicology, 84104 Bratislava, Slovakia; katarina.valachova@savba.sk (K.V.); Karol.Svik@savba.sk (K.S.); ladislav.soltes@savba.sk (L.S.); 2 Department of Pathology, St. Elizabeth Cancer Institute Hospital, 81250 Bratislava, Slovakia; csaba.biro1675@gmail.com; 3 School of Engineering, Bernal Institute, University of Limerick, V94T9PX Limerick, Ireland; 4 National Agricultural and Food Centre–RIAP Nitra, 95141 Luzianky, Slovakia; rastislav.jurcik@nppc.sk (R.J.); lubomir.ondruska@nppc.sk (L.O.)

**Keywords:** free/^•^OH radicals, rotational viscometry, skin injuries, thiol compounds

## Abstract

A high-molecular weight hyaluronan is oxidatively degraded by Cu(II) ions and ascorbate—the so called Weissberger biogenic oxidative system—which is one of the most potent generators of reactive oxygen species, namely ^•^OH radicals. Ergothioneine, hercynine, or histidine were loaded into chitosan/hyaluronan composite membranes to examine their effect on skin wound healing in ischemic rabbits. We also explored the ability of ergothioneine, hercynine, or histidine to inhibit hyaluronan degradation. Rotational viscometry showed that ergothioneine decreased the degree of hyaluronan radical degradation in a dose-dependent manner. While histidine was shown to be potent in scavenging ^•^OH radicals, however, hercynine was ineffective. *In vivo* results showed that the addition of each investigated agent to chitosan/hyaluronan membranes contributed to a more potent treatment of ischemic skin wounds in rabbits compared to untreated animals and animals treated only with chitosan/hyaluronan membranes.

## 1. Introduction

Hyaluronan (HA) is a glycosaminoglycan consisting of repeating d-glucuronic acid and *N*-acetyl-d-glucosamine disaccharide units. HA is abundant in the human body with over 50% present in the skin [1]. The residence time of HA is short, exhibiting a half-life of 1–2 days [2]. In skin exposed to UV irradiation, HA may act as a scavenger of free radicals and an antioxidant in physiological conditions [3,4]. The high-molar-mass HA is also a component of both the cartilage and synovial fluid. The degradation of high-molar-mass HA occurs under inflammation and oxidative stress and is accompanied by the loss of viscoelastic properties of synovial fluid [5]. In osteoarthritis, articular applications of sterile HA solutions, termed viscosupplementation, diminish pain and disability and thus enhance the function of joints and decrease cartilage degradation [6].

Wound healing is usually divided into several sequential phases, in which HA plays a key role, that overlap such as homeostasis, inflammation, granulation, tissue formation, and tissue remodeling [7]. In the inflammatory phase, HA binds to fibrinogen to begin clotting. In the proliferative phase, HA draws fibroblasts to the wound site. It creates cushioning and structural organization within the extracellular matrix. Furthermore, HA can stimulate metalloproteinases for angiogenesis, while promoting keratinocyte migration and proliferation. In the remodeling (granulation) phase, HA contributes to normal and pathological scarring [8]. It is known that increased concentrations of HA in the serum indicate several inflammatory skin diseases, such as psoriasis, progressive systemic sclerosis, and dermatomyositis [9].

Chitosan (Ch) is a β-1,4-linked polymer of glucosamine and *N*-acetylglucosamine. It is a derivative of chitin (poly-*N*-acetylglucosamine) [10]. Preparations of chitosan of various molar masses and degrees of deacetylation have attracted much attention due to their potentially beneficial biological properties [11,12]. The chitosan ability to bind with red blood cells allows rapid clotting of the blood, and it was approved in the USA for use in bandages comprising hemostatic agents [13]. Furthermore, chitosan modulates the functions of inflammatory cells and subsequently promotes granulation and organization. As a semipermeable biological dressing, it maintains a sterile wound exudate beneath a dry scab, prevents dehydration and contamination of the wound. Chitosan is antimicrobial due to its ability to destabilize the outer membrane of Gram-negative bacteria and its ability to permeate the microbial plasma membrane [14] and has been used to deliver bacteriocin [15].

**Figure 1:**
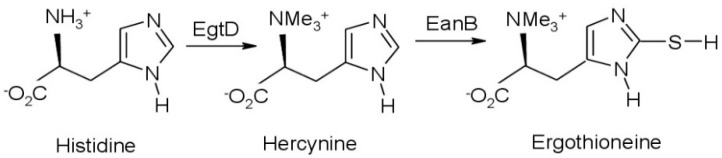
Biosynthetic pathway of ergothioneine under anaerobic conditions adapted from Valachova et al. [16]: The enzyme EgtD converts the amino acid histidine into hercynine (Me, methyl group). The enzyme EanB catalyzes the synthesis of ergothioneine directly from hercynine in the presence of a sulphur donor under anaerobic conditions.

Ergothioneine is a sulfur-containing amino acid discovered a century ago in the rye ergot. The only organisms, which synthesize it are bacteria of the order *Actinomycetales* (for example, mycobacteria) and fungi including *Lentinus edodes* (shiitake), *Pleurotus ostreatus*, and *Pleurotus eryngii*. These microbes synthesize ergothioneine from histidine using an intermediate hercynine, as shown in Figure 1.

Mammals acquire ergothioneine exclusively through diet. Ergothioneine is tautomeric and is present in the thione form in neutral aqueous solutions. Under *in vitro* conditions, ergothioneine inhibits the formation of ^•^OH radicals, O_2_^•−^, ^1^O_2_ production, lipid peroxidation, and peroxynitrite oxidative damage. It protects the skin against UV light [17,18]. It can bind to transition metal ions such as Fe^2+^/Fe^3+^ and Cu^2+^/Cu^+^ in forms unable to catalyze redox reactions and protects cells from apoptosis. Humans have the ergothioneine transporter protein (OCTN1). Human tissues, e.g., liver, kidney, central nervous system, bone marrow, and red blood cells receive ergothioneine from dietary sources up to millimolar concentrations [19,20,21,22,23,24]. Studies in animals and humans have found no toxicity or adverse effects to be associated with ergothioneine administration even at high doses. This is due to the presence of OCTN1 [25]. Ergothioneine is commonly used in cosmetics and skin care products [17]. Numerous investigations report that ergothioneine modulate inflammation, protect against acute respiratory diseases, neuronal damage, lung and liver fibrosis, mitigate damage to lungs, kidneys, liver, gastrointestinal tract and prevent endothelial dysfunction, which are symptoms attributed to a new type of corona virus [26]. Sotgia et al. [27] showed for the first time that hercynine, the main biosynthetic precursor and oxidative metabolite of ergothioneine, was detectable and measurable in beverages such as tea, coffee, beer, and wine. They propose hercynine to be a possible contributor to the antioxidant activity of ergothioneine.

Histidine acts as ^•^OH radical and singlet oxygen scavenger. The daily requirement for histidine is 8 to 12 mg/kg of body weight per day in adults. Free histidine and histidine incorporated into peptides and proteins are essential components of the antioxidative defense system. In plasma and other body fluids, histidine coexists with cysteine and other thiol compounds [28]. Histidine acts on metal regulation and chelates different metal ions such as cobalt(II), nickel(II), copper(II), zinc(II) cadmium(II), and iron(II) [29]. Histidine permeates the skin to reach the full dermis, down to the keratinocytes, where it renders several restorative functions [30]. Tan et al. [31] showed a beneficial effect of oral histidine in the treatment of adult patients with atopic dermatitis. Rothkopf [32] reported that histidine supplementation produced symptomatic improvement in the case of severe, treatment-resistant eczema in patients.

In our previous studies, we prepared Ch/HA membranes loaded with edaravone, which were characterized and examined *in vivo* in rats [33]. In 2018, Tamer et al. [34] prepared and characterized membranes composed of chitosan, HA, and MitoQ, whereas the addition of MitoQ had a beneficial effect on the structure of membranes and their application on skin wounds of rats and ears of ischemic rabbits contributed to a more rapid healing of the wounds. Similarly, tiopronin and captopril added to Ch/HA membranes were potent to facilitate healing of lacerations in ischemic ears of rabbits [35]. The Ch/HA membranes loaded with glutathione showed to be more beneficial in the treatment of skin wounds in rats than in untreated rats and rats treated only with Ch/HA membranes [36].

The aim of this study is to examine the ability of ergothioneine, hercynine, and histidine to inhibit reactive oxygen species-induced hyaluronan degradation and to determine their effect on healing skin wounds in ischemic rabbits when incorporated in hyaluronan chitosan membranes. We used ergothioneine since it is a special molecule—it is not toxic at high levels, its half-life is 1 month, and it is not oxidized. It protects the skin from UV light but it was not examined as a component of wound dressings. Histidine and hercynine are components used for the synthesis of ergothioneine in bacteria.

## 2. Materials and Methods

### 2.1. Materials

HA (M_w_ = 1.69 MDa, M_w_/M_n_ = 1.63) was purchased from Lifecore Biomedical Inc., Chaska, MN, USA. Chitosan (molar mass range: 100,000–300,000 Da) was obtained from ACROS Organics™, part of Thermo Fisher Scientific, Waltham, MA, USA. NaOH, ethanol, formaldehyde solution, trypane blue, formalin, haematoxylin, and eosin were purchased from Sigma-Aldrich, St. Louis, MO, USA. CuCl_2_⋅H_2_O p.a. and NaCl p.a. were purchased from Slavus Ltd., Bratislava, Slovakia. Ascorbic acid was from Merck KGaA, Darmstadt, Germany. De-ionized high-purity grade water, with conductivity of ≤0.055 µS/cm, was made using the TKA water purification system (Water Purification Systems GmbH, Niederelbert, Germany).

Twelve crossbred 6-month-old male rabbits HIL (2.5 ± 0.5 kg) from the Department of Toxicology and Breeding of Laboratory Animals at the Centre of Experimental Medicine in Dobra Voda, Slovakia were used.

### 2.2. Preparation of Stock and Working Solutions

The working solutions of the HA samples (16 mg) were prepared in the dark at room temperature in 0.15 mol/L NaCl in two steps: The first, 4.0 mL of the solvent was added, then 3.90 or 3.85 mL of the solvent was added after 6 h. Stock solutions of ergothioneine, hercynine, and histidine at a concentration of 16 mmol/L and their dilutions to 8.0, 1.6, and 0.32 mmol/L were made in 0.15 mol/L of NaCl. Stock solutions of ascorbic acid (16 mmol/L) and cupric chloride (160 µmol/L) were made in 0.15 mol/L of NaCl.

### 2.3. Hyaluronan Degradation

First, HA degradation was induced by an oxidative system comprising CuCl_2_ (1.0 μmol/L) and ascorbic acid (100 μmol/L). The procedure was as follows: A volume of 50 μL CuCl_2_ solution was added to the HA solution (7.90 mL), and stirred for 30 s. The mixture was maintained unstirred for 7.5 min at room temperature. Then, 50 μL of ascorbic acid solution was added to the HA solution, stirred for 30 s and followed by an immediate addition into the Teflon^®^ cup reservoir for viscometric measurements. The above procedure was repeated with 50 μL of ergothioneine, hercynine, or histidine (16, 8.0, or 1.6 mmol/L) added to the HA solution before the HA degradation begins or 1 h later.

### 2.4. Rotational Viscometry

Dynamic viscosity of the reaction mixture (8 mL) containing HA (2 mg/mL), ascorbate (100 μmol/L) along with Cu(II) ions (1 μmol/L) in the absence and presence of the examined compounds (10, 50, and 100 μmol/L) was reported by a Brookfield LVDV-II+PRO digital rotational viscometer (Brookfield Engineering Labs., Middleboro, MA, USA). The parameters of the measurement were: Temperature 25.0 ± 0.1 °C, shear rate of 237.6 s^−1^, 180 rpm, data report every 3 min within 5 h [37,38].

### 2.5. Preparation of Composite Membranes

Chitosan (0.5 g) was dissolved in 20 mL of aqueous acetic acid (2%, *v*/*v*). Hyaluronan (50 mg) was dissolved overnight in 5 mL of water. Both solutions were then mixed together, and 1 mL of the aqueous stock of ergothioneine, histidine, or hercynine solution (1.47 mg/mL) were admixed into the homogeneous Ch/HA solution. Next, 1 mL of glycerol as a plasticizer was added into the three component solution. This solution was then cast on a Petri dish and the solvent was allowed to evaporate at room temperature over 72 h. The dry membrane, separated from the Petri dish, was rinsed for approx. 1 min in a 1 mol/L NaOH solution to remove traces of acetic acid. The membrane was then washed for approx. 2 min in distilled water. Finally, the wet membrane was spread out and left to dry for several days at room temperature. Two types of membranes were prepared: Control Ch/HA membranes and Ch/HA membranes loaded with ergothioneine, hercynine, or histidine. Membranes were sterilized by spraying them with 80% ethanol, and dried.

### 2.6. Skin Wound Healing in Ischemic Rabbits

Experiments were approved by the ethical committee of the Institute of Experimental Pharmacology and Toxicology in Bratislava, Slovakia (SK UCH 04018), followed by the State Veterinary and Food Administration in Bratislava, Slovakia (2908-3/2020-220). Ischemic wounds on rabbits’ ears were performed according to DiPietro’s and Burns’s method [39]. Inside of each rabbit’s ear, two lacerations with a size of ca. 1 × 1 cm and a complete removal of skin tissue were performed. Rabbits were divided into three groups: First group−control (wound was covered with bandage only); second group—animals treated with the Ch/HA membrane only; and third group—animals treated with Ch/HA/ergothioneine, Ch/HA/hercynine, or Ch/HA/histidine membrane. Post operation animals underwent standard care. Animals were administered analgesics during the study. Rabbits were maintained individually in cages with an area of 4200 cm^2^ in daily 12 h light-dark cycles. Animal wounds were covered with dehydrated membranes immediately after the primary treatment of wounds. Each membrane was moisturized in saline and disinfected with 80% ethanol. Membranes were renewed after 3, 6, 9, and 12 days. Wounds were only washed with saline and in treated animals membranes were fastened to wounds with standard plasters. All wounds were bandaged. Untreated animals and the efficacy of Ch/HA and Ch/HA/ergothioneine, Ch/HA/hercynine, or Ch/HA/histidine membranes on the healing of skin wounds were evaluated through the measurment of wound area. To statistically evaluate the performance of the membranes an ANOVA test was carried out. Results are shown as the average and standard deviation for each group of animals.

## 3. Results and Discussion

**Figure 2:**
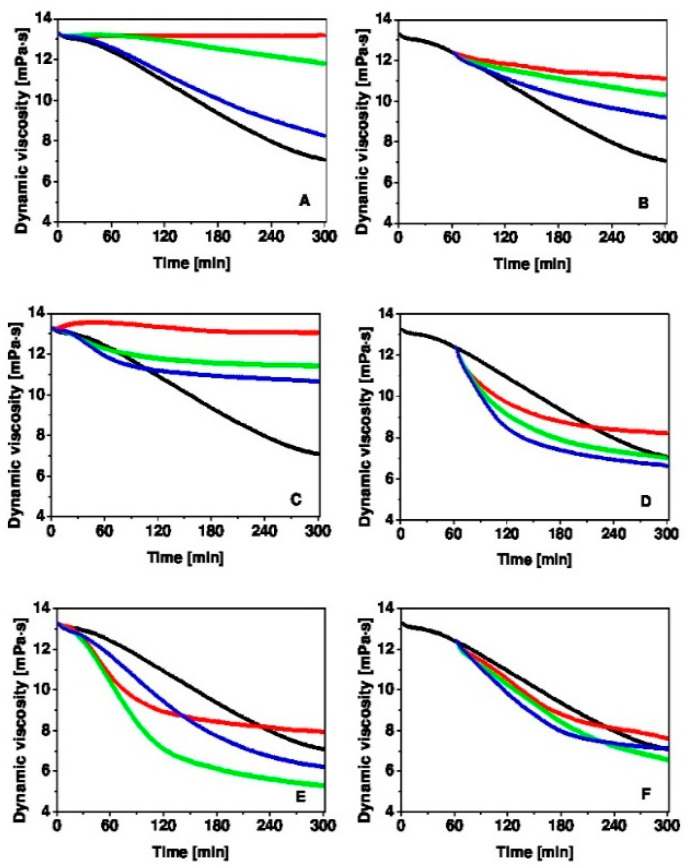
Time-dependent changes in dynamic viscosity of the HA solution exposed to 1 µmol/L Cu(II) ions and 100 µmol/L ascorbic acid (black curve) and after the addition of ergothioneine (**A**,**B**), histidine (**C**,**D**), hercynine (**E**,**F**) before HA degradation begins (left panels) and 1 h later (right panels). The compounds were added at concentrations: 100 µmol/L (red curve), 50 µmol/L (green curve), 10 µmol/L (blue curve).

Figure 2 shows the predispostion of the HA macromolecule to degradation initiated by Cu(II) ions (1 µmol/L) and ascorbate (100 µmol/L) with a viscosity decrease of 6.1 mPa⋅s within 5 h (black curve, the reference). The addition of ergothioneine (panel A) at a concentration of 100 µmol/L results in retardation of the ^•^OH radical-induced degradation of HA (red curve). Ergothioneine at a concentration of 50 µmol/L protects HA from degradation for 1 h, after that there is a slow decrease in the dynamic viscosity of the HA solution (green curve). A decrease in the ergothioneine concentration to 10 µmol/L (blue curve) facilitates HA degradation, however, it is less rapid than the reference (black curve). As shown in Figure 2B, ergothioneine also dose-dependently protects HA from free radical degradation, when added to the HA reaction mixture 1 h later.

Figure 2C shows that histidine at its highest concentration of 100 µmol/L significantly inhibits the degradation of HA (red curve). Histidine retards HA degradation also at concentrations of 50 and 10 µmol/L (green and blue curve, respectively). As shown in Figure 2D, histidine dose-dependently decreases the rate of HA degradation, when added to the reaction mixture 1 h later.

However, hercynine does not exhibit a concentration-dependent inhibition of the free radical-induced degradation of HA when added to the HA oxidative system before HA degradation was initiated (Figure 2E) and 1 h later (Figure 2F). Unlike ergothioneine, hercynine has no effect on the degradation of high-molar-mass HA. This is attributed to the functional group –SH in ergothioneine, which allows ergothioneine to be a potent donor of ^•^H, which interacts with ^•^OH and retards the initial phase of free radical degradation of HA. The OCTN1 protein is a selective transporter of ergothioneine, and we postulate that this may allow the possibility of a controlled release of ergothioneine into the blood stream.

**Figure 3:**
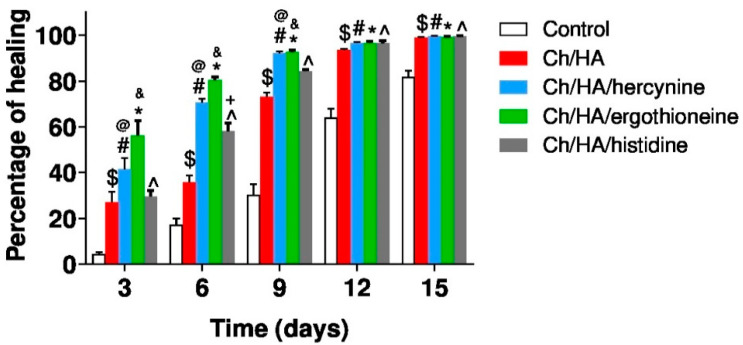
Profiles of the wound healing in ischemic rabbits, when the wound was not treated (control), the wound treated with the chitosan/HA (Ch/HA) membrane only (red), loaded with hercynine (blue), ergothioneine (green), or histidine (grey). N = 6. ^$^ indicates a significant difference between the control and the Ch/HA membrane at *p* ≤ 0.05. ^#^ indicates a significant difference between the control and hercynine loaded membrane at *p* ≤ 0.05. * indicates a significant difference between the control and ergothioneine loaded membrane at *p* ≤ 0.05. ^^^ indicates a significant difference between the control and histidine loaded membrane. ^@^ Indicates a significant difference between the Ch/HA membrane and hercynine loaded membrane and & indicates a significant difference between the Ch/HA membrane and ergothioneine loaded membrane. ^+^ Indicates a significant difference between the Ch/HA membrane and histidine loaded membrane.

The results in Figure 3 illustrate the percentage of healing of the untreated skin wound and the skin wound treated with the Ch/HA membrane alone and loaded with ergothioneine, hercynine, or histidine. On day 3 in control animals (untreated, white column) the potency in healing injured skin is 4%. The treatment of the wounds with the Ch/HA membrane (red column) enhanced the effect of healing up to 27%. The most potent examined Ch/HA membrane is shown to be the one with the addition of ergothioneine (green column). The efficiency in wound healing reached 56.2%. The least effective substance is histidine at 30%. On day 6, the healing increases in both control animals (white) and animals treated with Ch/HA membranes (red). However, the addition of the examined substances enhances the rate of healing, which is again the most potent for wounds treated with the Ch/HA/ergothioneine membrane (green). The wounds heal up to 80% efficacy, while the addition of histidine has the least benefit.

A more significant healing of skin wounds is seen on day 9, when the percentage of healing increased up to 92% after loading Ch/HA composite membranes with ergothioneine (green column) and hercynine (blue column). The treatment of skin wounds with the Ch/HA composite membranes loaded with histidine reached 81% (grey column). In contrast, the percentage of healing skin wound with the Ch/HA membrane increases to 73% and in untreated animals the healing reaches only 28% efficacy (white column).

On day 12, the percentage of healing skin wounds treated with the Ch/HA membranes loaded with the all examined substances reaches about 96%, which is slightly better than the treatment of the skin wound with only the Ch/HA membrane (red column). While on day 15, all animals treated with the Ch/HA membranes reach almost full healing compared to the untreated animals (white column), which exhibit an 82% level of healing. The statistical significance of these results are reported in Figure 3.

**Figure 4:**
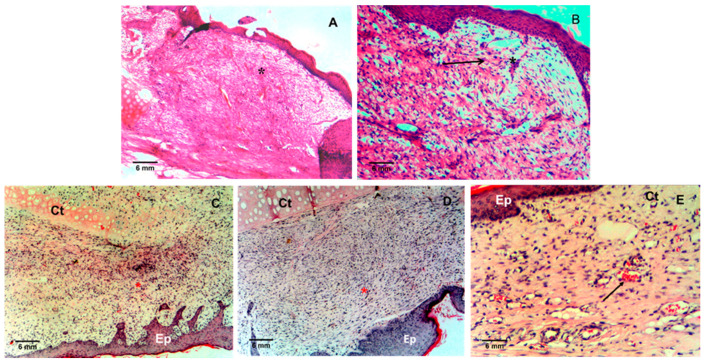
Histograms of the rabbit ischemic ear tissues from: Control experiment (**A**), after treatment with Ch/HA membranes (**B**), after treatment with the Ch/HA composite membrane loaded with histidine (**C**), hercynine (**D**), or ergothioneine (**E**). Ct: Cartilage; Ep: Epidermis.

Figure 4A illustrates the tissue of the untreated animals (control group), which was in a phase of inflammation/proliferation. In the histogram, one can see the vascular maturating granular tissue (*), where a less amount of histocytes, leukocytes, hyperemic capillaries with perivascular bleeding and perpendicular distribution of fibroblasts prevail. Further, in animals treated with Ch/HA membranes (Figure 4B), it is seen that within 15 days the tissue is in a proliferative phase. There is an obvious maturating granular tissue, which is assumed to be composed particularly of leukocytes, macrophages, myxoid changes of the stroma, plasmocytes, and fibroblasts due to the presence of acid mucopolysaccharides. There are activated fibroblasts with the formation of collagen fibres (→) and newly formed veins (*).

Figure 4C illustrates the hypocelullar nonspecific granular tissue with a loss of inflammatory elements (*). The wound treated with Ch/HA/histidine composite membranes is in a phase of proliferation/remodeling. The result of the treatment of the wound with Ch/HA/hercynine composite membranes illustrates the periphery of the wound in ischemic area, as shown in Figure 4D. Changes are in a phase of remodulation. The presence of fibroblasts/myofibroblasts is obvious. There is an absence of polymorphonuclear granulocytes and macrophages (*).

The result of the treatment of the wound with Ch/HA/ergothioneine composite membranes displays the remodulation phase of wound healing, as shown in Figure 4E. The wound is composed of hypocellular nonspecific granular tissue with the prevalence of myofibroblasts and fibrous collagen.

Our study is the first, where ergothioneine has been explored as a component of wound dressings used for the treatment of injured skin, and is the subject of a recent patent application [40]. Ergothioneine is an established and very potent antioxidant species, which functions in the organism as a bulwark and a potent cytoprotective compound [19].

## 4. Conclusions

In conclusion, ergothioneine and histidine are potent in attenuating free radical HA degradation. In contrast, hercynine was ineffective. Results of *in vivo* experiments show that the addition of ergothioneine and histidine to Ch/HA membranes contributes to a quicker rate of healing of ischemic skin wounds in rabbits, with ergothioneine performing the best of all, especially during the earlier phases of healing. It can be speculated that the incorporation of ergothioneine and its subsequent release from biopolymeric membranes allows its transport to the site of inflammation via the blood stream and this is mediated by the OCTN1 protein. These findings are relevant as the skin contains a high content of this ergothioneine.

## Data Availability

The data presented in this study is openly available.
